# Low-Dose Amphotericin B and Murine Dialyzable Spleen Extracts Protect against Systemic *Candida* Infection in Mice

**DOI:** 10.1155/2013/194064

**Published:** 2013-09-10

**Authors:** F. Robledo-Ávila, M. Pérez-Tapia, A. Limón-Flores, L. Pavon, R. Hernández-Pando, I. Wong-Baeza, G. González-González, C. Tovar, S. Estrada-Parra, I. Estrada-García

**Affiliations:** ^1^Department of Immunology, National School of Biological Sciences (ENCB), National Polytechnic Institute (IPN), 11340 Mexico City, DF, Mexico; ^2^Bioprocesses Development and Research Unit (UDIBI), National School of Biological Sciences (ENCB), National Polytechnic Institute (IPN), 11340 Mexico City, DF, Mexico; ^3^Department of Immunology, Faculty of Medicine, Autonomous University of Nuevo León (UANL), 64460 San Nicolás de los Garza, NL, Mexico; ^4^Laboratory of Psychoimmunology, National Institute of Psychiatry “Ramón de la Fuente”, 14080 Mexico City, DF, Mexico; ^5^Department of Pathology, National Institute of Medical Sciences and Nutrition “Salvador Zubirán”, 14000 Mexico City, DF, Mexico; ^6^Department of Microbiology, Faculty of Medicine, Autonomous University of Nuevo León (UANL), 64460 San Nicolás de los Garza, NL, Mexico; ^7^Department of Microbiology, National School of Biological Sciences (ENCB), National Polytechnic Institute (IPN), 11340 Mexico City, DF, Mexico

## Abstract

*Candida albicans* causes opportunistic systemic infections with high mortality (30%–50%). Despite significant nephrotoxicity, amphotericin (AmB) is still used for the treatment of this serious fungal infection. Therefore, alternative treatments are urgently needed. Dialyzable leukocyte extracts have been used successfully to treat patients with mucocutaneous candidiasis, but their effectiveness in systemic candidiasis has not been evaluated. In this study, low-dose AmB (0.1 mg/kg) plus 10 pg of murine dialyzable spleen extracts (mDSE) were tested in a systemic candidiasis mouse model. Survival, tissue fungal burden, kidney damage, kidney cytokines, and serum levels of IL-6 and hepcidin were evaluated. Our results showed that the combined treatment of low-dose AmB plus mDSE improved survival and reduced kidney fungal burden and histopathology; these effects correlated with increased kidney concentration of IFN-**γ** and TGF-**β**1, decreased levels of TNF-**α**, IL-6, and IL-10, as well as high levels of systemic IL-6 and hepcidin. Low-dose AmB and mDSE synergized to clear the infectious agent and reduced tissue damage, confirming the efficacy of a low dose of AmB, which might decrease the risk of drug toxicity. Further studies are necessary to explore these findings and its implications in future therapeutic approaches.

## 1. Introduction

Opportunistic mycoses are infections caused by saprophytic or commensal fungi in hosts in which the normal microbiota has been altered by antibiotic treatments, in hosts with disrupted anatomic barriers, or in immunosuppressed hosts [1]. *Candida albicans* is an ubiquitous, dimorphic fungus that colonizes the skin, gastrointestinal tract, and oral and vaginal mucosa of immunocompetent individuals without causing disease [2], but it can cause opportunistic mucocutaneous and systemic infections (with a mortality of 30–50%), particularly in hospitalized patients [1, 3, 4]. 

Neutrophils and macrophages play a central role in the immune response against *C. albicans*, and decreased numbers of these cells correlate with increased tissue fungal burden and mortality [5]. Neutrophils and macrophages are activated through several pattern-recognition receptors, including Toll-like receptor (TLR) 2, TLR4, TLR9, C-type lectin receptor, dectin-1, dectin-2, DC-SIGN, mincle, galectin-3, SCARF1/CD36, and NLRP3. Recognition of *C. albicans* by dectin-1 triggers CARD9 signalling, and mutations in CARD9 lead to chronic mucocutaneous candidiasis and invasive *Candida* infections in humans [6]. The proinflammatory cytokines TNF-*α* and IL-6 are also critical for the immune response against *C. albicans* [5].

The recognition of *C. albicans* yeasts by TLR4 leads to the production of IL-12 and a Th1 response [7], while the recognition of hyphae by dectin-1 and dectin-2 triggers the production of IL-23 and a Th17 response [7]. The recognition of hyphae by TLR2 is associated with the induction of a Th2 response [7], which is not protective but could be involved in the regulation of the inflammatory response, since mice that lack TLR2 are resistant to systemic candidiasis [8, 9].

Several antifungal drugs are available for the treatment of *C. albicans* infections, including amphotericin B (AmB), 5-fluorocytosine, fluconazole, itraconazole, voriconazole, posaconazole, and ravuconazole, but their use is limited because of their toxicity and their low efficacy rates [10]. Echinocandins are a new class of antifungal drugs that inhibit the synthesis of *β*-glucan in *C. albicans* cell wall. They are effective against most isolates of *Candida* spp. and they are less toxic than other antifungal drugs, but they are expensive [11]. Dialyzable leukocytes extracts (DLE) are low molecular weight-dialyzable peptides from immune cells, which have immunomodulatory activities [12]. DLE have been used in clinical settings for the treatment of several diseases, including herpes zoster, herpes simplex type I, herpetic keratitis, atopic dermatitis, osteosarcoma, tuberculosis, asthma, post-herpetic neuritis, anergic coccidioidomycosis, leishmaniasis, toxoplasmosis, sinusitis, pharyngitis, and otitis media [13]. Intramuscular DLE have been used successfully to treat mucocutaneous candidiasis in humans [13–17]. 

In a previous report, we showed that experimental murine tuberculosis could be successfully treated with a combination of murine dialyzable spleen extracts (mDSE) and conventional chemotherapy [18]. Here, we established an animal model of systemic candidiasis, where the efficacy of low-dose AmB supplemented with mDSE could be assessed. We evaluated the effects of the combined treatment on survival, tissue fungal burden, tissue damage, kidney cytokines, and hepcidin and IL-6 serum levels. We provide evidence that the combination of low-dose AmB plus mDSE is effective for the control of murine systemic candidiasis.

## 2. Materials and Methods

### 2.1. Ethics Statement

This study was carried out in strict accordance with the recommendations from the Guide for the Care and Use of Laboratory Animals (NOM-062-ZOO-1999) of the “National Technical Consultation Council for Animal Wellbeing” (CONASA), Ministry of Health, Mexican Government. The protocol was approved by the “Investigation Committee for the Transference Factor Project (CIPFT)” of the National School of Biological Sciences, IPN (Authorization no. IB-10-004). 

The followup of all the experimental groups was documented daily by trained animal caretakers. In the experiments where survival was being evaluated, mice were separated from their experimental group and humanely sacrificed by cervical dislocation, when signs of distress (significant weight lost, fever, piloerection, and hyperventilation) were detected. When organs were collected, the mice were previously euthanized by cervical dislocation.

### 2.2. Experimental Model of Systemic Candidiasis

*C. albicans* 07-387 (*Ca*07-387) was isolated from a patient with systemic candidiasis at the UANL. *Ca*07-387 was cultured at 37°C for 18 h on a rotating drum in Sabouraud medium (Difco, Sparks, MD, USA) and frozen at 5 × 10^6^ CFU/mL in 30% glycerol. For each experiment, a vial was thawed and yeasts were cultured to exponential phase. 

To establish the best infecting dose, groups of 5 to 10 female BALB/c mice (4-5 weeks old, 14–16 g) were infected intravenously (i.v.) with different amounts of *Ca*07-387 blastospores in 0.1 mL of sterile saline solution and observed for 30 days. (older mice, 8−12 weeks old, were resistant to the infection). The 5 × 10^5^ dose was chosen for the rest of experiments. Groups of 5 infected mice were treated with different concentrations of AmB (Sigma-Aldrich, St. Louis, MO, USA) in 0.1 mL of water (i.v.), on days 2, 3, 4, 5, and 6 after infection. 

### 2.3. Preparation of Murine Dialyzable Spleen Extracts (mDSE) and Treatment of Infected Mice

mDSE was obtained from 10 healthy adult BALB/c mice (10–12 weeks old). Spleen cell suspensions were disrupted by five cycles of freezing and thawing (−20°C/37°C). Lysates were subjected to three cycles of filtration (2,300 g for 15 min) using Centricon centrifugal filter devices (Millipore, Billerica, MA, USA), with a nominal molecular weight limit of 10 KDa. Filtrates (mDSE) were tested for endotoxin (Gel clot LAL method, Charles River Endosafe, Charleston, SC, USA), sterility, and total peptide content (bicinchoninic acid assay, Pierce Biotechnology, Rockford, IL, USA). The mDSE preparation was sterile and had <0.125 endotoxin units/mL and 96 *μ*g peptides/mL. Infected mice were injected intramuscularly (i.m.) with 10 pg of mDSE, alone or in combination with 0.1 mg/kg AmB (i.v.), on days 2, 3, 4, 5, and 6 after infection.

*Ca*07-387 infected mice were divided into four experimental groups (each with 20–25 mice). Each group received a different treatment: 0.1 mg/kg AmB, 10 pg mDSE, 0.1 mg/kg AmB, and 10 pg mDSE, or saline. Three mice from each group were euthanized on days 2, 10, 15, and 30 after infection. Kidneys, spleens, livers, and brains were weighed, macerated, diluted with saline, and cultured overnight on Sabouraud dextrose agar (Difco) to determine tissue fungal burden. Blood samples were taken from these mice by facial vein puncture, in accordance with the Official Mexican Guidelines (NOM-062-ZOO-1999), and serum aliquots were frozen at −20°C.

### 2.4. Histopathological Analysis of Tissue Samples

The kidneys, spleen, liver, and brain of mice were taken at the indicated time points and immediately fixed by immersion in 10% formaldehyde/PBS, dehydrated in ethylic alcohol, embedded in paraffin, sectioned, and stained with haematoxylin and eosin (HE), or Gomori-Grocott methenamine silver nitrate staining method (GG). Slides were analyzed under light microscopy (Olympus BX40).

### 2.5. Hepcidin and Cytokine Quantification

Hepcidin was quantified in serum samples by ELISA. The assay was set using mouse hepcidin (HEPC11-P, Alpha Diagnostic International, San Antonio, TX, USA), rabbit anti-mouse hepcidin antibody (HEPC11-A, Alpha Diagnostic International), and a protein A-HRP conjugate (Bio-Rad, Hercules, CA, USA). IFN-*γ*, TNF-*α*, IL-2, IL-4, IL-6, IL-10, and IL-17A were quantified in macerated kidneys and serum samples using Cytometric Bead Array multiplexed bead-based immunoassays (BD Biosciences, San Jose, CA, USA); 2,500 events were acquired for each sample in a FACSAria flow cytometer (BD). Data were analysed using FlowJo software (TreeStar, San Carlos, CA, USA). TGF-*β*1 was quantified with an ELISA kit (e-Biosciences, San Diego, CA, USA).

### 2.6. Statistics

Survival curves were analyzed with Kaplan-Meier log-rank test, and CFU and cytokines were analyzed with two-way ANOVA and Bonferroni posttest.

## 3. Results

### 3.1. Treatment with Low-Dose AmB Plus mDSE Increases Survival in Mice with Systemic Candidiasis

**Figure 1:**
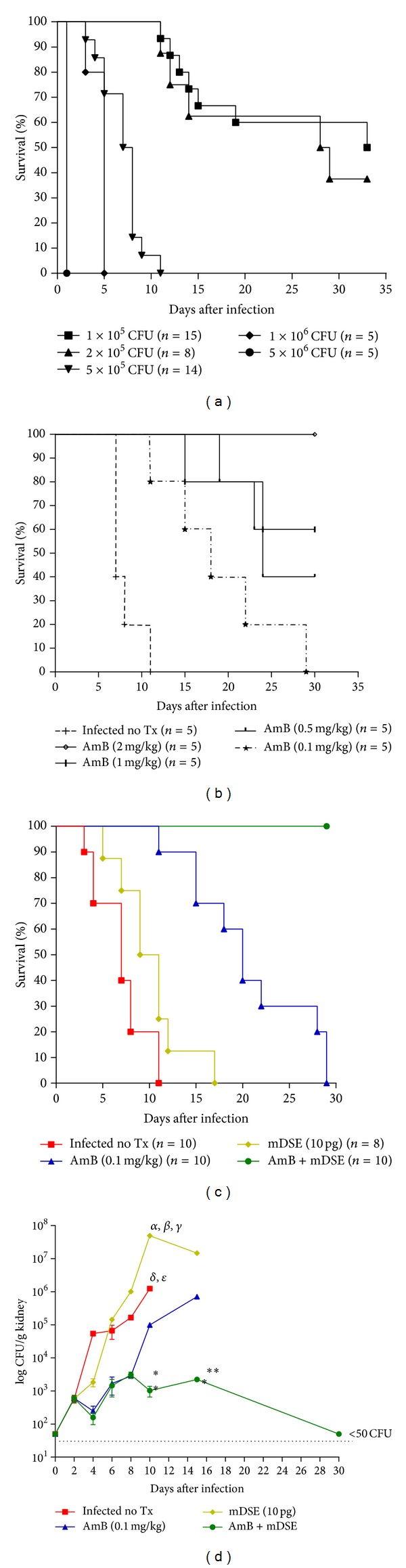
Effect of low-dose AmB and mDSE on murine systemic candidiasis. (a) Mice were infected with the indicated amounts of *Ca*07-387 blastospores and were observed for 30 days. Data from two independent experiments were used. (b) Different concentrations of AmB were administered to mice that had received 5 × 10^5^ CFU of *Ca*07-387. (c) Mice that had received 5 × 10^5^ CFU of *Ca*07-387 were treated with 10 pg of mDSE, alone or in combination with 0.1 mg/kg of AmB. Data from two different experiments were used. (d) Kidney fungal burdens (CFU/g) each point represents mean and SD of three mice. In (d), the dotted line indicates the assay detection limit (<50 CFU) (**P < 0.01; ***P < 0.001, AmB versus AmB + mDSE), (*α* = *P* < 0.001, mDSE versus Infected no Tx), (*β* = *P* < 0.001, mDSE versus AmB), (*γ* = *P* < 0.001, mDSE versus AmB + mDSE), (*δ* = *P* < 0.05, Infected no Tx versus AmB), (*ε* = *P* < 0.05, Infected no Tx versus AmB + mDSE).

All mice infected i.v. with 5 × 10^6^, 1 × 10^6^, and 5 × 10^5^ CFU presented piloerection, fever, and significant weight loss (data not shown) and died after 1, 5 and 11 days, respectively. In contrast, mice infected with 2 × 10^5^ and 1 × 10^5^ CFU showed 40% and 60% of survival after 30 days, respectively (Figure 1(a)). The surviving animals did not show any signs of infection at this time. We used 5 × 10^5^ CFU for all further experiments, because this dose provided sufficient time to test the effect of different treatments. The administration of 0.1 mg/kg of AmB (low-dose AmB) to infected mice did not prevent their death but extended their lifespan to 28 days (Figure 1(b)). For this reason, we chose this dose to evaluate the effect of mDSE. A high dose of AmB (2 mg/kg) was required to prevent death of all infected animals (Figure 1(b)).

The administration of 10 pg of mDSE alone did not affect the survival of mice infected with 5 × 10^5^ CFU. In contrast, the administration of low-dose AmB in combination with 10 pg of mDSE produced 100% survival (Figure 1(c)). Mice treated with low-dose AmB plus mDSE showed a significant decrease in kidney fungal burden since day 10 after infection, when compared to mice treated only with mDSE or low-dose AmB (Figure 1(d)). The AmB group controlled kidney fungal burden until day 8 (2 days after the last administration of AmB), when the fungi started to grow exponentially. Fungal burdens in the spleens, livers and brains were 2-log lower than in the kidneys, and no differences in the fungal burden of these organs were observed between the groups (data not shown). 

### 3.2. Treatment with AmB Plus mDSE Ameliorates the Histopathology Induced by Systemic Candidiasis

**Figure 2:**
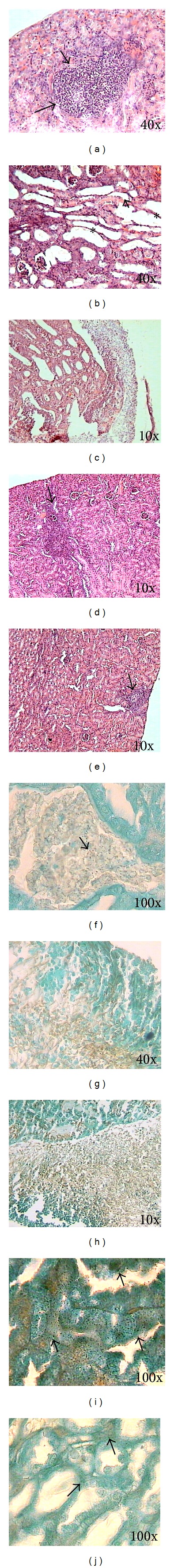
Representative histological features of kidneys from mice with systemic candidiasis. Mice were infected with 5 × 10^5^ CFU of *Ca*07-387 and were left untreated (a), (b), (f), and (g) or were treated with 10 pg mDSE (c) and (h), 0.1 mg/kg AmB (d) and (i), or mDSE and AmB (e) and (j). Kidney sections were taken on days 2 (a) and (f) and 10 (b)–(e), (g)–(j) after infection and stained with HE (a)–(e) or with GG (f)–(j).

Mice infected with *Ca*07-387 showed progressive kidney damage: after 2 days of infection, well-defined abscesses with abundant neutrophils (arrows, Figure 2(a)) and yeasts (arrow, Figure 2(f)) were observed in the cortical and medullar regions. These abscesses were larger after 10 days of infection; numerous proximal convoluted tubules had necrotic and detached epithelial cells (arrow, Figure 2(b)), some tubules were completely denuded (asterisks, Figure 2(b)), and abundant hyphae were present (Figure 2(g)).

The histopathological changes in mice infected with *Ca*07-387 and treated with mDSE were similar to those of untreated mice; their kidneys showed necrosis in the pelvic area (Figure 2(c)) and abundant hyphae (Figure 2(h)). The kidneys from mice infected with *Ca*07-387 and treated with AmB showed considerable fibrotic scars (arrow, Figure 2(d)), abundant yeast in the tubular regions (arrows, Figure 2(i)), and no evidence of tubular damage. Interestingly, the kidneys from mice infected with *Ca*07-387 and treated with mDSE and AmB showed limited scar tissue (arrow, Figure 2(e)), no signs of tubular damage, mild inflammation, and few yeast (arrows, Figure 2(j)). No significant histological differences were observed in spleens, livers, and brains from these four experimental groups.

### 3.3. Effects of Low-Dose AmB and mDSE on Kidney Cytokines, Systemic IL-6, and Hepcidin on Mice with Systemic Candidiasis

**Figure 3:**
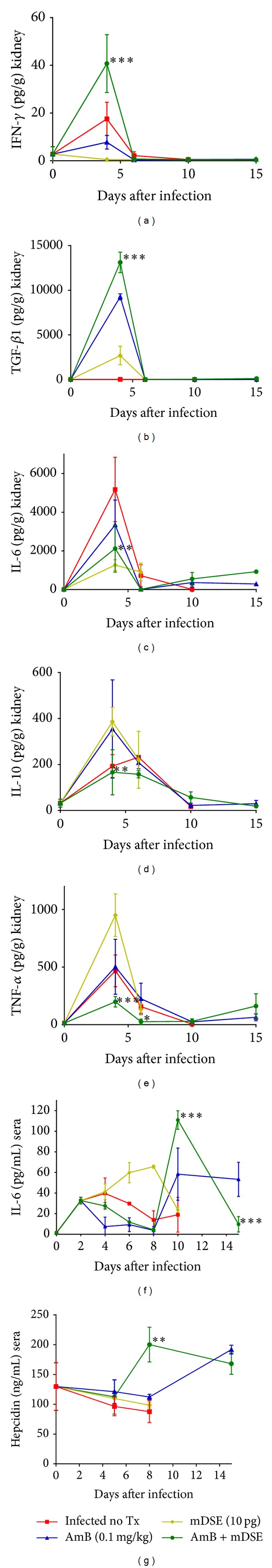
Effects of low-dose AmB and mDSE on kidney and serum cytokines in systemic candidiasis. Mice were infected with 5 × 10^5^ CFU of *Ca*07-387 and treated with 10 pg of mDSE in combination with 0.1 mg/kg of AmB. (a) IFN-*γ*, (b) TGF-*β*1, (c) IL-6, (d) IL-10, and (e) TNF-*α* were measured in macerated kidneys. Each point represents mean and SD of three mice. (f) Serum IL-6 and (g) serum hepcidin in each experimental group: each point represents mean and SD of three mice. **P < 0.01, ***P < 0.001, AmB versus AmB + mDSE.

The combined treatment modulated the levels of kidney cytokines in mice with systemic candidiasis on day 4 after infection (2 days after treatment initiation). IFN-*γ* and TGF-*β*1 concentrations were significantly increased (Figures 3(a) and 3(b)), while IL-6, IL-10, and TNF-*α* were decreased in comparison with the AmB group (Figures 3(c), 3(d), and 3(e)). No differences were found in IL-2, IL-4, or IL-17A levels (data not shown). Serum IL-6 concentration was significantly higher (*P* < 0.001) on day 10 after infection in mice that received the combined treatment, when compared with mice treated with AmB alone (Figure 3(f)). No differences were detected for serum IFN-*γ*, TGF-*β*1, TNF-*α*, IL-2, IL-4, IL-10, or IL-17A (data not shown). 

Serum hepcidin was significantly higher on day 8 in mice that received the combined treatment, compared with mice that were treated with low-dose AmB alone; in the latter group, serum hepcidin increased its concentration only after day 15 (Figure 3(g)). 

## 4. Discussion

The intravenous infection model of systemic candidiasis recapitulates several features of the human disease [5]. In this model, fungal cells are delivered directly to the bloodstream, and infection is controlled in most organs (including the liver and the spleen), but not in the kidneys and (in cases of high inoculum levels), the brain. Mice die of progressive sepsis and develop renal failure, whose severity correlates with kidney fungal burden [5]. The experimental model of systemic candidiasis that we established was in line with the previously reported models.

We used this model to evaluate the efficacy of low-dose AmB supplemented with mDSE for the treatment of systemic candidiasis. Although several drugs, including echinocandins, are effective for the treatment of *C. albicans* infections, AmB is still used in many clinical settings, and the use of low-dose AmB is desirable because of the drug's toxicity. In previous studies, DLE were used in combination with antifungal drugs to treat human candidiasis [14–17], and the combination was more effective in controlling the infection than the drug alone. Our results indicate that the combined treatment of low-dose AmB plus mDSE significantly improved the effect of the drug, promoting an efficient control of the *Ca*07-387 strain and reducing tissue damage. Since AmB has nephrotoxic effects in humans [19], this combination would reduce the risk of toxicity associated with the administration of high doses of this drug (up to 5 mg/kg/day for 7 days in patients). 

We found increased levels of IFN-*γ* and TGF-*β*1 in the kidneys of mice that received the combined treatment, compared to mice that received AmB alone. Previous studies demonstrated that Th1 responses mediated by IFN-*γ* resolved *C. albicans* infection [20, 21] by inducing nitric oxide and ROS production [22]. TGF-*β*1 limits the damage caused by excessive inflammation and promotes tissue regeneration [23]. Mice that were treated with AmB alone had higher concentrations of the pro-inflammatory cytokines TNF-*α* and IL-6 in their kidneys, but lower concentrations of IFN-*γ*, compared to mice that were treated with AmB plus mDSE. These results suggest that mice treated only with AmB have increased inflammation but lower protection from *C. albicans*. 

The elimination of fungi in our experimental model correlated with high levels of serum hepcidin, which is a peptide hormone and a type II acute phase protein produced by the liver in response to iron overload and inflammatory stimuli, particularly IL-6 [24]. Hepcidin regulates the transcription of several inflammatory mediators: it binds to ferroportin, induces the activation of Jak2 and Stat3, increases the levels of SOCS3, and thus decreases the signal transduction of TLRs and cytokine receptors [25]. Hepcidin is also an antimicrobial peptide that can be detected in blood and urine, and it has direct fungicidal activity against *C. glabrata *isolates *in vitro* [26].

Serum hepcidin was significantly higher on day 8 in mice that received AmB plus mDSE, compared with mice that only received AmB; in the latter group, serum hepcidin increased its concentration only after day 15. The increased levels of serum IL-6 in mice that received the combined treatment could account for hepcidin production [24]. Mice that were treated with mDSE alone also had increased levels of serum IL-6; however, these mice did not produce hepcidin, possibly because of their high concentrations of kidney TNF-*α*, a negative regulator of hepcidin expression [24]. These high concentrations of kidney TNF-*α* might also explain why infected mice treated with mDSE alone had a higher kidney fungal burden than infected but untreated mice: the inflammation induced by this cytokine could cause tissue damage (necrosis), which would promote hyphae growth. IFN-*γ* upregulates hepcidin expression [27], so the increased amounts of IFN-*γ* in the kidneys of mice with the combined treatment could also contribute to the early production of this peptide.

Our results showed that the combination of low-dose AmB and mDSE cleared the infectious agent and at the same time reduced inflammation-associated tissue damage. During systemic candidiasis, kidney infection is associated with neutrophil infiltration [5]. The kidney damage could be caused directly by the infection, because there is a strong correlation between kidney fungal burden and serum creatinine levels [28]. However, a decreased recruitment of neutrophils to the kidney was associated with improved renal function, decreased inflammatory kidney damage, and increased survival, but it had no effect on kidney fungal burden [29]. This suggests that neutrophils (and their inflammatory mediators) are in part responsible of the tissue damage. In line with these observations, it was reported that, in patients who develop chronic disseminated candidiasis during neutrophil recovery after intensive chemotherapy, treatment with corticosteroids in addition to antifungals caused an improvement of the clinical symptoms and the resolution of the inflammatory response [30].

An inherent limitation of our study is that the intravenous infection model of systemic candidiasis represents the late stages of the disease, when the fungal cells are already in the bloodstream. It does not address the early stages of the disease (i.e., translocation of *C. albicans* from the gut), which would explain how *C. albicans* reaches the blood. However, we provide evidence that indicates that the combination of low-dose AmB with human DLE could have appropriate efficacy and safety as a treatment for systemic candidiasis.
